# A Review of the Effects of Ipsilateral or Contralateral Vaccine Boosting on the Adaptive Immune Response

**DOI:** 10.3390/vaccines13121225

**Published:** 2025-12-04

**Authors:** Amal Naji, Hana M. El Sahly, Jennifer A. Whitaker

**Affiliations:** 1 Department of Medicine, Section of Infectious Diseases, Baylor College of Medicine, One Baylor Plaza, Houston, TX 77030, USA; amal.naji@bcm.edu; 2 Departments of Molecular Virology and Microbiology and Medicine, Section of Infectious Diseases, Baylor College of Medicine, One Baylor Plaza, Houston, TX 77030, USA; hana.elsahly@bcm.edu

**Keywords:** vaccine, ipsilateral, contralateral, immune response

## Abstract

Vaccines have been pivotal in reducing the incidence and severity of infectious diseases, improving population health, and lowering mortality rates globally. While substantial progress has been made in optimizing vaccine formulations, adjuvants, and schedules, comparatively less attention has been given to how the site of vaccination may influence immunologic outcomes. This review examines the impact of the administration of prime and booster vaccine doses in the same (ipsilateral) versus the opposite arms (contralateral) on vaccine immunogenicity. We review animal model and human studies evaluating the impact of ipsilateral versus contralateral COVID-19 and non-COVID-19 vaccine boosting on immunologic outcomes with a focus on the germinal center response, antibody production, and T cell activation. While some studies suggest that ipsilateral administration may enhance the quality of germinal center B cell responses and antibody magnitude, data across different studies have been inconsistent. Relatively few studies have compared ipsilateral versus contralateral boosting, and differences in study design and outcomes have limited the ability to draw conclusions as to whether one is superior to the other. This review highlights a noteworthy and underexplored area in vaccinology and the need for future research to clarify whether ipsilateral/contralateral boosting strategies matter. To answer this question, high-quality, randomized controlled trials evaluating different types of vaccines that consider immunologic mechanisms, capture key time points and appropriate specimens, and evaluate early and long-term immunogenicity endpoints are required.

## 1. Introduction

Vaccines have been crucial in significantly reducing the burden of deadly diseases over the years [1,2]. They have been incorporated in healthcare infrastructure, reduced mortality rates, and lowered healthcare costs [3,4]. Immunization programs around the world have played a transformative role in diminishing the impact of diseases like smallpox, polio, tetanus, and measles, to the extent that many people today scarcely recall the severe consequences these diseases once had [3,4,5].

Recently, the COVID-19 pandemic caused widespread morbidity and mortality and disrupted the socioeconomic fabric of society. It was not until COVID-19 vaccines were introduced that the virus’ toll on public health was effectively limited, and life gradually returned to near normalcy [6,7].

The immunogenicity and efficacy of different classes of vaccines are influenced by a variety of factors including the type of infectious agent, vaccine type, and the characteristics of the host, such as age, sex, medical comorbidities, genetics, gut microbiota, and immune history [8,9]. Significant efforts are underway to enhance the effectiveness of vaccines against pathogens that cause significant morbidity and mortality through the use of adjuvants that allow dose sparing, new vaccine platforms that might induce better mucosal immunity, and evaluation of new routes of administration [9,10,11,12].

Understanding vaccines’ mechanism of action is crucial for the development and improvement of vaccines. Most vaccines against infectious pathogens work by generating neutralizing antibodies [13]. Other vaccines are designed to stimulate a cytotoxic T cell response [14,15]. To achieve an effective immune response, a few vaccines, like the yellow fever vaccine, are administered as a single dose. In contrast, most vaccines use a prime-boost approach in people who are naïve to the specific vaccination, requiring two or more doses to reach optimal immunogenicity and effectiveness [16].

Little attention is paid to the choice of arm when administering the booster vaccine dose. The decision is typically left to the discretion of either the patient or the person administering the booster. However, recently, researchers have begun to investigate how the immunogenicity and efficacy of booster vaccines are influenced by whether they are administered in the same arm or the contralateral arm as the first vaccine dose [17]. There is limited evidence in the literature regarding the impact of the choice of arm on vaccine immunogenicity. In this review, we summarize the evidence regarding the impact of administering prime and booster vaccine doses in the same (ipsilateral) versus the contralateral arm on vaccine immunogenicity and efficacy and outline future directions for research in this insufficiently evaluated area of vaccinology.

## 2. Immune Response to Vaccine in Draining Lymph Node

**Figure 1:**
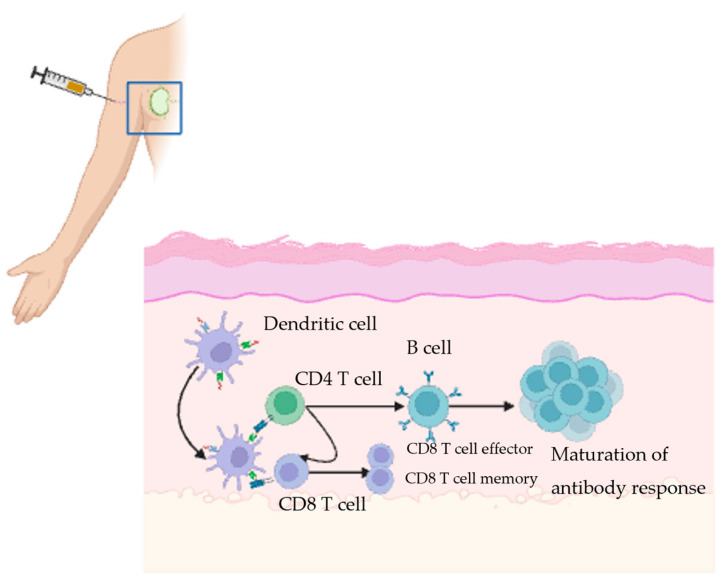
Created in BioRender.com Illustration of the adaptive immune response triggered by a vaccine. Vaccine antigens are delivered at the injection site, where antigen presenting cells (APCs) attach to them. They then travel to nearby lymph nodes. In the lymph nodes, T and B cells are activated, leading to antibody production and generation of plasma cells. The antibodies then circulate to neutralize the pathogen. The memory cells provide long-lasting protection against future exposures.

The study of the immune response to vaccines has been facilitated by the fine needle aspiration technique, which has enabled monitoring of the immune response elicited in the adjacent draining lymphoid tissue following vaccination. When a vaccine is administered, antigen-presenting cells (APCs), usually dendritic cells, take up antigens and then travel to the draining lymph node. Signals displayed on major histocompatibility complex class II molecules are then recognized by the T-cell receptor (TCR) on T cells, which subsequently leads to their activation. T-follicular helper cells and specialized CD4+ T cells carry a variety of signals to the B cells in the germinal center (GC) (Figure 1). These signals trigger a GC reaction, where the activated B cells undergo somatic hypermutation. Shortly after, the high affinity B cells are positively selected and subsequently differentiate into plasma cells and memory B cells. The plasma cells produce antibodies that can potentially neutralize the pathogen. Memory B-cells serve as a second line of defense by rapidly generating a burst of plasma cells that produce antibodies upon re-exposure to the pathogen [18,19,20,21]. CD8+ T-cells also play an important role in the immune response to some pathogens and vaccines. CD8+ T cells frequently recognize proteins on the surface of APCs and then become activated. Upon activation, CD8+ T cells employ two primary mechanisms to exert their effect. The first mechanism involves lysing infected cells by secreting effector molecules such as perforin and granzyme B, which create pores in the target cells. The second mechanism involves the death of the infected cells through the interaction of the Fas ligand and Fas receptor [22,23,24]. Therefore, the immune response to the vaccine frequently involves a collaboration between B cells and T cells (Figure 1).

## 3. Effect of Administering the Vaccine Boosters in the Ipsilateral Versus Contralateral Arm on Germinal Center Response

The primary aim of administering a booster vaccine is to generate a durable and effective immune response that can last for months to years. GCs in the draining lymph nodes are crucial to this process because they play an essential role in enhancing the immune response to vaccines. Within these centers, B cells are believed to drive the peak antibody response, typically starting around the seventh day after vaccination [25,26]. The importance of GCs in vaccine response was highlighted in a study comparing lymph node responses in severely ill COVID-19 patients to those in vaccinated individuals. This study revealed significant deficiencies in GCs within the peri-bronchial lymph nodes of severely ill patients compared to the axillary lymph nodes of vaccinated individuals. The findings indicated substantial disruption of CD21+ follicular dendritic cell networks and a marked decrease in BCL6+ cells, including both GC B cells and T follicular helper (Tfh) cells in individuals with severe COVID-19 infection. This disruption was evident in both primary and secondary follicles, suggesting widespread alterations in lymph node architecture among COVID-19 patients. In contrast, mRNA vaccination was associated with follicular hyperplasia and well-developed GC structures [27].

Research on the GC response to various booster vaccines has been conducted over many years. While a single vaccine dose activates B cells in the GC, a more pronounced response with higher antibody titer is typically observed only after booster doses are administered. These additional doses help to amplify the initial immune activation, leading to an increased quantity of antibodies and a stronger overall defense against the targeted pathogen [28].

Following the introduction of the COVID-19 vaccine, studies have shown varying results regarding the impact of the site of vaccine prime and booster dose administration on the magnitude of the immune response. In mouse models, administering COVID-19 booster doses resulted in an eightfold increase in the frequency of activated GC B cells. When comparing ipsilateral and contralateral vaccinations, both groups had comparable neutralizing and binding antibody responses to the vaccine antigen, and a substantial 100–200-fold increase in the number of GC B cells in the draining lymph nodes, while non-draining lymph nodes showed only a modest 2–5 fold increase. Despite this disparity, the overall GC B cell response was similar in both groups. Ipsilateral vaccines led to the activation of pre-existing GCs in the draining lymph nodes, while contralateral boosts resulted in the activation of de novo GCs. Ultimately, these mechanisms resulted in similar frequencies of total activated GC centers between the two groups [29] (Table 1). However, another study comparing ipsilateral and contralateral COVID-19 vaccines in mice by Jiang et al. reported that the quality of the immune response differed between the two groups, with the ipsilateral group showing (1) a higher number of receptor-binding domain (RBD)-specific GC B cells, (2) expansion from pre-existing GC B cells, (3) Day 2 and Day 4 increase in RBD-specific plasmablasts, and (4) Day 4 enhanced T cell receptor signaling in Tfh cells. These findings indicate subtle differences in the immune response to vaccination between the ipsilateral and contralateral groups that are more pronounced in the early days post vaccination, and the significance of these findings remains to be determined [30] (Table 1).

## 4. Impact of Administering Vaccine Boosters in the Ipsilateral Versus Contralateral Arm on Antibody Response

The observed differences in the GC response prompted researchers to investigate the impact of vaccination site on antibody response, which is driven by activated B cells that eventually differentiate into plasma cells [30,31,32].

A study by Jacobson et al. examined the antibody response in draining versus nondraining lymph nodes in rabbits following a single dose of the diphtheria toxoid (DT) or bovine gamma globulin (BGG) vaccine. The vaccines were administered in the left pad, and the researchers then assessed the antibody response in the draining (left, ipsilateral) and nondraining (right, contralateral) lymph nodes. Antibody titers were detected as early as day 7 in the ipsilateral draining lymph nodes, while a response in the contralateral non-draining lymph nodes was delayed until day 9. Lower antibody titers were observed in the non-draining lymph nodes and this difference persisted for three months. Researchers suggested that the disparity was due to the presence of persistent antigens in the cortex of the draining lymph nodes, whereas access to the antigen in the contralateral lymph nodes was minimal [31] (Table 2).

These findings on localized immune responses parallel historical studies on pre-exposure rabies vaccines and booster doses in humans, which looked at the response to the booster vaccine. Individuals who received a booster dose experienced a fourfold increase in serological antibody titers compared to those who received a single dose. However, significant variations were seen in seroconversion rates, ranging from 30% to 98%. This variability was not attributed to differences in vaccine batches or timing, but rather to the vaccination technique employed. Notably, individuals who received all four doses intradermally in the same arm demonstrated a 92% response rate compared to only 68% of those who received two doses intradermally in each arm, suggesting that repeated stimulation of the same regional lymph nodes enhances immune response [32] (Table 2).

A more recent open-label randomized controlled study examined the administration of routine infant immunizations, including the diphtheria-tetanus-acellular pertussis vaccine (DTaP), inactivated polio (IP), *Haemophilus influenzae* type b (Hib), and 13-valent pneumococcal vaccines (PCV-13) in consistent versus alternating legs. Infants in the consistent limb group received the DTaP-IP-Hib combined vaccine at 2, 3, and 4 months of age, and the pneumococcal conjugate vaccine (PCV13) at 2, 4, and 12 months, all administered to the right leg. Infants in the alternating limb group received DTaP-IPV-Hib in the left leg at 2 months and in the right leg at 3 and 4 months; and PCV13 in the left leg at 2 months, in the right leg at 4 months, and in the left arm at 12 months. All infants in both groups received the combined Hib and capsular group C Neisseria meningitidis tetanus toxoid conjugate vaccine (Hib-MenC-TT), administered in the left leg at 12 months. The geometric mean concentrations (GMC) of anti-Hib anti-polyribosylribitol phosphate IgG were significantly lower in the consistent limb at 5 and 12 months. The GMC of anti-tetanus toxoid antibodies were also significantly lower in the consistent arm at 13 and 24 months. However, there was no difference in the anti-pneumococcal IgG antibody titers between the two groups. At 24 months, almost 100% of the participants had a geometric mean concentration of anti-pneumococcal antibodies that exceeded the established protection levels (Table 2). Overall, these findings were inconsistent with the results of the rabies study. The interval between vaccine doses can possibly explain the inconsistent results since, for example, the pneumococcal vaccine doses were given 2 months apart while the rabies vaccines were administered just 1 week apart. The authors postulated that alternating injection sites may promote the recruitment of a greater number of draining lymph nodes, which could enhance the immune response [33].

**Table 2:** Summary of animal and human studies evaluating the site of vaccine administration on immune response to non-COVID vaccines.

Study	Model	Primary End Point	Time Evaluated Post-Vaccination	Outcomes
**Animal Studies**				
Jacobson et al., 1969 [31]	Rabbit	Antibody response To DT and BCG vaccines	7 days; 2–3 weeks; 4 weeks, 3–4 months	-Ipsilateral lymph nodes showed an antibody response at day 7 -The contralateral lymph nodes showed a response at day 9 -The antibody response in the contralateral lymph nodes was significantly lower over time
Kuraoka et al., 2022 [34]	Mice	Levels of serum IgG, secondary GCBC response to influenza vaccine	4–5 weeks; 8–10 weeks; 12–14 weeks	-Similar IgG and plasmacyte responses between groups -Higher antigen-specific clonal IgG in ipsilateral group -More highly mutated B cells in ipsilateral boost group -More efficient recruitment of primary GC B cells in ipsilateral group
**Human Studies**				
Peck et al., 1964 [32]	Adults	Antibody response to rabies vaccine	52 days	-Higher antibody response in the ipsilateral group compared to the contralateral group
Iro et al., 2015 [33]	6–12 weeks old infants	Geometric mean concentration of anti-tetanus toxoid, anti-pneumococcal and anti-*H influenzae* type B antibodies	1 months, 5 months, 12 months, 24 months	-Anti-Hib GMTs lower in consistent limb than alternating legs at 5 months and 12 months -Anti-tetanus toxoid IgG were lower in consistent than alternating legs at 13 months and 24 months -Anti-pneumococcal IgG means were similar between both groups at all time points

DT = Diphtheria toxoid; BGG = Bovine gamma globulin; GC = germinal center; anti-Hib = anti-*Haemophilus influenzae* B titers; GMT = geometric mean titers.

More recently, following the COVID-19 pandemic, researchers investigated the impact of COVID-19 mRNA vaccine administration site on the antibody response. A study by Jiang et al., conducted in mice, showed an increase in RBD-GC B cells in the ipsilateral group, and they assessed the antibody response 9 days and 19 weeks after the booster vaccination. Their focus was on RBD-IgG and spike-specific IgG titers, and they found no differences in these titers. These findings led them to investigate antibody affinity, where they observed a temporary increase in the affinity of RBD-IgG antibodies at the 9-day time point in the ipsilateral group. However, this effect disappeared by the 19-week time point (Table 1). Additionally, there was no change in the affinity of spike-specific antibodies at any time. Therefore, the increase in GCs following ipsilateral vaccination did not translate into a corresponding change in the antibody response at later time points [30].

Human studies evaluating the effect of alternating COVID-19 vaccine doses in ipsilateral and contralateral arms were observational in design and yielded conflicting results [17]. In a study by Ziegler et al., 303 individuals who received the second dose of the priming series of mRNA COVID-19 vaccine in the contralateral (156 individuals) or ipsilateral (147 individuals) arms were enrolled, and antibody responses were assessed at 2 weeks post dose 2. A statistically significant difference was observed in the neutralizing antibody response, with the contralateral group exhibiting significantly lower neutralizing activity compared to the ipsilateral group (65% vs. 69%, *p* = 0.024) [17]. On the other hand, in an observational study by Fazli et al., individuals who received the second COVID-19 vaccination in the contralateral arm had significantly higher binding and neutralizing antibody responses lasting up to 1-year post-vaccination. This discrepancy between the two human studies is possibly related to the differences in the timing of the sample collection [35]. Ziegler et al. assessed the antibody response at 2 weeks, which is relatively early in the immune response maturation process. At this point, it is possible that the preformed GCs at the site of initial vaccination contributed to a more robust response in those receiving ipsilateral boosting [17]. Both studies were observational, and it is possible that some other unmeasured confounders contributed to some of the differences [17,35] (Table 3).

In a recent study, the co-administration of COVID-19 and influenza vaccines were evaluated in ipsilateral versus contralateral arms. The hemagglutination inhibition antibody titers and SARS-CoV-2 antibody responses were comparable between the groups [36]. A retrospective cohort study conducted in Israel among Clalit Health service members who received the BNT162b2 vaccine during a period between 2020–2021 evaluated the effect of ipsilateral versus contralateral arm dose of the second BNT162b2 vaccine dose on the primary outcome of PCR-confirmed SARS-CoV-2 infection in the 28 days after second dose of vaccine. The study reported an unadjusted odds ratio (OR) for the occurrence of the primary endpoint in the ipsilateral versus contralateral recipients of 0.77 (95% CI, 0.68–0.87, *p* < 0.001) and adjusted OR (adjusting for age and sex) of 0.83 (95% CI, 0.73–0.84, *p* = 0.004). This study had the limitations of being retrospective, nonrandomized, and evaluating a short period of time after completion of the second vaccine dose.

## 5. Effect of Site of Administration on the T Cell Response

T cell responses also play an important role in the immune response to many pathogens and vaccines. Therefore, it is crucial to identify the optimal vaccination approach, including the site of booster vaccine administration, to enhance T cell responses [29]. In the observational study by Ziegler et al., no differences were observed in the total percentage of the CD4 T cell response between the two groups. However, spike-specific CD8 T cell levels were significantly lower in the contralateral group compared to the ipsilateral group [17]. These results were inconsistent with findings from studies in mice, which showed no difference in spike-specific CD8 T cells between the ipsilateral and contralateral groups [17,29,30]. As a result, there is considerable variability in the findings of various studies investigating the connection between the site of vaccine administration and the resulting T cell response. While some studies suggest that the location of vaccine delivery can influence the magnitude or quality of the T cell-mediated immune response, others report no significant effect, leading to inconsistent and sometimes contradictory conclusions across the literature.

## 6. Summary and Future Directions

This review explored animal model and human studies that evaluated the effect ipsilateral versus contralateral boosting of COVID-19 and non-COVID-19 vaccines on immunologic outcomes. The two studies that evaluated the impact of booster site administration on germinal center responses were both mouse-model studies evaluating COVID-19 vaccines. While ipsilateral boosting led to a greater increase in the GC B cells in the draining lymph nodes, contralateral boosting resulted in the activation of de novo GCs, and the total number of activated GCs was similar between the groups with comparable neutralizing and binding-antibody responses [29]. In contrast, the other study demonstrated a higher number of RBC-specific GC B cells, greater expansion in the RBC-specific plasmablasts, and T cell receptor signaling in Tfh cells at early time points after vaccination (Days 2 and 4) in the ipsilateral vaccination group [30]. As these studies evaluated different immunologic endpoints and timepoints, it is difficult to compare them. It may be that the early differences noted between ipsilateral/contralateral vaccination groups do not result in differences in antibody magnitude, quality, or persistence at later time points. Human studies of COVID-19 mRNA vaccines showed disparate results with one reporting higher neutralizing antibody titers in the ipsilateral group at 15 days after vaccination [17] and the other reporting higher spike-specific antibodies in the contralateral group at 2.5 weeks, 8 months, and 1.1 years after vaccination [35]. It may be that the differences observed were related to the differences in the time points evaluated.

Animal model studies evaluating the effect of ipsilateral/contralateral non-COVID-19 vaccines are very limited. A rabbit model for DT and BCG vaccines demonstrated superior antibody responses [31] and a mouse model for influenza vaccine demonstrated superior B cell responses [34] with ipsilateral immunization.

Human studies evaluating the effect of booster site administration reveal disparate results, with one study of rabies vaccination showing higher antibody titers in the ipsilateral group [32] and the other showing lower titers in the ipsilateral groups for anti-Hib, tetanus toxoid IgG, and similar results between groups for pneumococcal IgG mean titers [33].

In conclusion, the site of booster vaccine site administration (ipsilateral vs. contralateral) may play a role in shaping the immune response, yet the data remain scarce and inconsistent. Differences in study design including the time from booster dose to blood sampling, differences in vaccine types, and study populations may have led to differing conclusions regarding the effects of ipsilateral versus contralateral vaccine boosting. Most studies have evaluated COVID-19 mRNA vaccines, and all have evaluated non-live vaccines. Live attenuated vaccines may have different effects on cellular immune responses and may be less subject to site administration. Therefore, it is important to consider the vaccine type when evaluating the effect of the vaccine booster site administration on cellular immune responses. Moreover, importantly and with one exception, all clinical studies were non-randomized.

In order to draw definitive conclusions as to whether the booster vaccine site administration impacts meaningful immunogenicity outcomes, future research is needed. First, different vaccine types need to be evaluated (inactivated, attenuated, subunit, mRNA vaccines). It could also be true that the vaccine adjuvant and vaccine administration route (subcutaneous, intramuscular, intradermal) could be affected differently by boost vaccine site administration. One cannot assume that the same effect will be observed across all classes of vaccines. Second, it may be easiest to study this effect for pathogens/vaccines to which the vaccine recipient is immunologically naïve. Third, it may be most meaningful to study pathogens for which there is a known correlate of protection and to evaluate immunologic outcomes that are clinically meaningful based upon the correlate of protection. Fourth, it is important to understand whether there is an effect on peak immunologic responses, in addition to long-term memory responses.

Animal model studies provide benefits in the ability to conduct lymph node sampling and germinal center studies that may be more challenging to conduct in human clinical trials. Studies that do find significant results regarding differences need to be replicated. Animal studies, preferably using large animal models, could also be accompanied by controlled challenge with the specific pathogen to detect impact on efficacy. Additionally, veterinary vaccine studies also provide an opportunity to explore this important question and evaluate meaningful immunologic outcomes, particularly when correlates of protection are known. If consistent and meaningful differences are detected in animal models, human clinical trials could be designed. These studies need to be optimally planned to eliminate bias, with the collection of appropriately timed sample collection essential to resolve these inconsistencies, to probe detailed differences in the human immune responses, and to help shed light on the mechanisms and significance of the differences.

## Figures and Tables

**Table 1:** Animal studies evaluating the effect of booster vaccine site administration on immune response to COVID vaccines.

Study	Model	Primary End Point	Time Evaluated Post-Vaccination	Outcomes
Ying et al., 2024 [29]	Mice	Impact of site of COVID vaccination on adaptive B and T cell immune response	1 day; 4 weeks	-Both booster locations induce similar IgG titers -Small variation in neutralizing activity of antibodies -Similar GCBC and T cell response in contralateral vs. ipsilateral groups
Jiang et al., 2024 [30]	Mice	GCBC and antibody response to COVID vaccines	2 days; 4 days; 9 days; 19 weeks	-Higher RBD-specific GCBC response in ipsilateral group -Higher high-affinity RBD-specific antibodies in ipsilateral boost group -More plasma cell differentiation of pre-existing GCBC in ipsilateral group

GCBC = germinal center B cell; RBD = receptor binding domain.

**Table 3:** Human studies evaluating the effect of booster vaccine site administration on immune response to COVID vaccines.

Study	Primary End Point	Time Evaluated Post- Vaccination	Outcomes
Ziegler et al., 2023 [17]	Levels of spike-specific IgG, IgG-avidity, neutralizing antibodies in response to COVID vaccine	15 days	-Spike-specific IgG levels were similar, but neutralizing activity was lower in the contralateral group -CD4 levels similar; higher CTLA-4 expression in contralateral group -Lower spike-specific CD8 T cells in the contralateral group
Fazli et al., 2024 [35]	Serological Response to COVID vaccine	2.5 weeks; 8 months; 1.1 years	-Higher spike-specific antibodies in the contralateral group
Pattison et al., 2024 [36]	Raw mean pre- and post-vaccination titers in response to COVID and influenza vaccine	22–55 days	-No difference in the immune response between the contralateral and ipsilateral group
Grupel et al., 2022 [37]	Positive RT-PCR in response to COVID vaccine	28 days (10 days after second vaccine dose)	-Positive SARS-CoV-2 PCR was more common in the contralateral group than the ipsilateral group -Adjustments for age and sex showed similar effect -COVID-19 related hospitalization and death was more frequently seen in the contralateral group

RT-PCR = reverse transcription polymerase chain reaction; cytotoxic T-lymphocyte antigen-4 (CTLA-4)

## Data Availability

No new data were created.

## Abbreviations

APC	Antigen-presenting cell
BGG	Bovin gamma globulin
DT	Diphtheria toxoid
DTaP	Diphtheria—tetanus- acellular pertussis
GC	Germinal center
GCBC	Germinal center B cell
GMC	geometric mean concentration
Hib	*Haemophilus influenzae* type b
Hib-MenC-TT	*Haemophilus influenzae* type b-capsular group C Neisseria meningitidis-tetanus toxoid conjugate
IP	Inactivated polio
PCV13	13-valent pneumococcal conjugate
RBD	Receptor binding domain
TCR	T cell receptor
Tfh	T follicular helper
